# A Dietary Test of Putative Deleterious Sterols for the Aphid *Myzus persicae*

**DOI:** 10.1371/journal.pone.0086256

**Published:** 2014-01-20

**Authors:** Sophie Bouvaine, Marie-Line Faure, Robert J. Grebenok, Spencer T. Behmer, Angela E. Douglas

**Affiliations:** 1 Department of Entomology, Cornell University, Ithaca, New York, United States of America; 2 Department of Molecular Biology and Genetics, Cornell University, Ithaca, New York, United States of America; 3 Department of Biology, Canisius College, Buffalo, New York, United States of America; 4 Department of Entomology, Texas A&M University, College Station, Texas, United States of America; University College Dublin, Ireland

## Abstract

The aphid *Myzus persicae* displays high mortality on tobacco plants bearing a transgene which results in the accumulation of the ketosteroids cholestan-3-one and cholest-4-en-3-one in the phloem sap. To test whether the ketosteroids are the basis of the plant resistance to the aphids, *M. persicae* were reared on chemically-defined diets with different steroid contents at 0.1–10 µg ml^−1^. Relative to sterol-free diet and dietary supplements of the two ketosteroids and two phytosterols, dietary cholesterol significantly extended aphid lifespan and increased fecundity at one or more dietary concentrations tested. Median lifespan was 50% lower on the diet supplemented with cholest-4-en-3-one than on the cholesterol-supplemented diet. Aphid feeding rate did not vary significantly across the treatments, indicative of no anti-feedant effect of any sterol/steroid. Aphids reared on diets containing equal amounts of cholesterol and cholest-4-en-3-one showed fecundity equivalent to aphids on diets containing only cholesterol. Aphids were reared on diets that reproduced the relative steroid abundance in the phloem sap of the control and modified tobacco plants, and their performance on the two diet formulations was broadly equivalent. We conclude that, at the concentrations tested, plant ketosteroids support weaker aphid performance than cholesterol, but do not cause acute toxicity to the aphids. In plants, the ketosteroids may act synergistically with plant factors absent from artificial diets but are unlikely to be solely responsible for resistance of modified tobacco plants.

## Introduction

Sterols are an essential constituent of eukaryotic membranes and contribute to other functions, notably hormones of animals (e.g. mammalian estrogens, insect ecdysteroids) and plants (e.g. brassinosteroids) [1], [2]. Despite the broadly equivalent function of sterols in animals and plants, the sterol profile differs between these two groups: animal sterols are generally dominated by cholesterol, while plants contain multiple sterols, known as phytosterols (e.g., sitosterol, stigmasterol), but generally very small amounts of cholesterol [3]. The composition of phytosterols varies among plant species, a trait that is of special significance for phytophagous insects for two linked reasons. First, insects, unlike most other animals, cannot synthesize sterols and are, therefore, dependent on a dietary supply of these nutrients; and, second, phytophagous insects vary in their capacity to utilize different phytosterols [4], [5]. A mismatch between the plant sterol content and the sterol utilization traits of a particular insect is predicted to confer plant resistance to the insect of interest [1] thereby limiting the plant range of the insect [4].

The basis of this study is an innovative approach to modify the phytosterol content of plants. Specifically, plants transformed with the bacterial gene *choM* (sterol oxidase) have a dramatically altered sterol profile, dominated by oxidized ketosteroids instead of phytosterols [6], [7]. Three lepidopterans [8], [9] and the aphid *Myzus persicae* [10] display depressed performance on tobacco plants bearing the *choM* transgene (“modified” plants) relative to plants transformed with the empty vector (“control” plants). The central importance of the ketosteroids in the resistance of modified plants against lepidopteran caterpillars is indicated by the very poor performance of the lepidopteran *Heliothis zea* on diet supplemented with cholest-3-one, a dominant ketosteroid in the modified plants, relative to diets with no sterol or sterols found in control plants [9].

**Figure 1:**
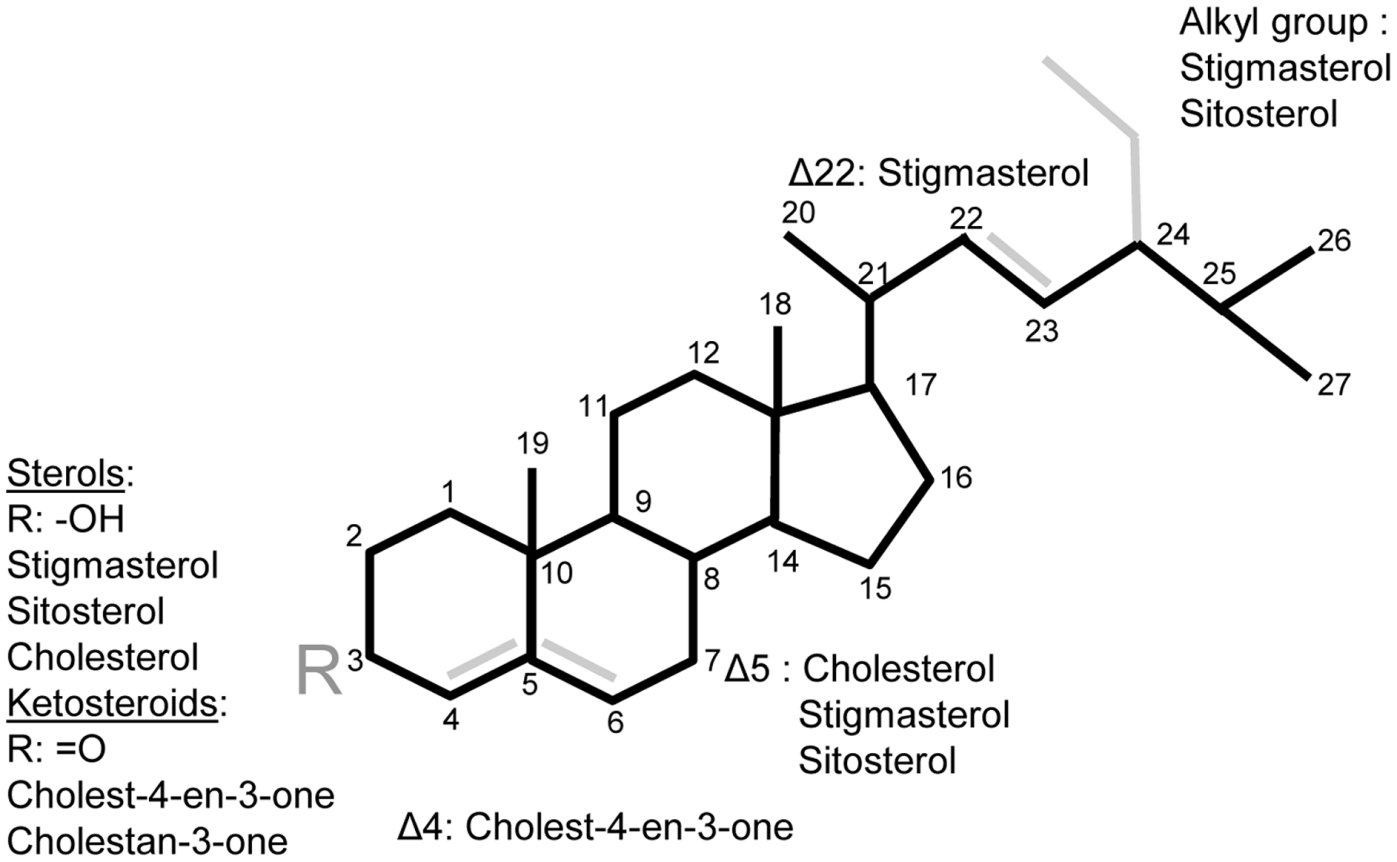
Structure of sterols and ketosteroids used in this study.

This study exploited the ease with which the aphid *M. persicae* can be reared on chemically-defined diets to investigate the effect of ketosteroids on aphid performance. The phloem sap of the modified tobacco plants is dominated by cholest-4-en-3-one and also contains cholestan-3-one (Figure 1); these two ketosteroids are undetectable in the control tobacco plants [10]. Given the negative effects of these two steroids on Lepidoptera, this study tested the hypothesis that the aphids would also display very high larval mortality and sharply curtailed fecundity on diets containing these ketosteroids, relative to diets with sterols detected in control plants (cholesterol and the phytosterols sitosterol and stigmasterol).

## Results

### Aphid Performance on Single-sterol Diets

To investigate the effect of the individual sterols and steroids on the performance of *M. persicae*, 2-day-old aphids born on sterol-free diets were transferred to chemically-defined diets supplemented with single sterols/steroids previously identified in the phloem sap of control or modified tobacco [9]. Each sterol was tested at one of three concentrations: 0.1, 1 and 10 µg ml^−1^. As a control, we also tested aphids on a sterol-free diet. The insects attached and fed readily on all the diets, and developed to adulthood over 10 days.

Fecundity, lifespan, and larval relative growth rate (Table 1) were similar across all treatments on the lowest dietary sterol/steroid concentration (0.1 µg ml^−1^). At the higher dietary concentrations, significant differences in fecundity and lifespan were observed (Table 1). At 1 µg dietary sterol ml^−1^, the highest fecundity was obtained with dietary cholesterol; this was also the only treatment for which dietary sterol ml^−1^, fecundity was equivalent on diets containing cholesterol, sitosterol and cholestan-3-one treatments, but the median fecundity was zero for aphids on sterol-free diet and diets containing 10 µg ml^−1^ of either the phytosterol stigmasterol or the ketosteroid cholest-4-en-3-one. The effect of diet on the lifespan of the aphids was also concentration-dependent. At 1 µg ml^−1^, dietary cholesterol promoted longer lifespan than other sterol/steroid supplements; and at 10 µg ml^−1^, lifespan did not differ among treatments, apart from the reduced lifespan of aphids on diets containing cholest-4-en-3-one. Median lifespan on the cholesterol and no-sterol control did not differ significantly across all three concentrations. Growth rate differences were observed between treatments with 1 ug ml^−1^, but not 10 ug ml^−1^ dietary sterol (Table 1). However, on the 1 ug ml^−1^ treatments, differences relative to cholesterol were all non-significant.

**Table 1:** Aphid performance on diets containing different dietary sterols.

Dietary sterol	Relative growth rate (g g^−1^ day^−1^) Median (range)			Lifespan (days) Median (range)			Number of reproducing aphids/total			Number of offspring aphid^−1^ Median (range)		
	0.1_µg ml^−1^	1 µg ml^−1^	10 µg ml^−1^	0.1_µg ml^−1^	1 µg ml^−1^	10 µg ml^−1^	0.1 µg ml^−1^	1 µg ml^−1^	10 µg ml^−1^	0.1 µg ml^−1^	1 µg ml^−1^	10 µg ml^−1^
None	0.258 (0.205–0.330)	0.273 (0.213–0.305)	0.275 (0.220–0.319)	24 (10–32)	23 (11–30)	23.5 (11–32)	4/10	4/10*	4/10*	0 (0–8)	0* (0–9)	0* (0–8)
Choles-terol	0.294 (0.185–0.344)	0.254 (0.215–0.287)	0.253 (0.149–0.309)	21 (13–31)	27 (17–33)	27.5 (23–30)	6/10	10/10	7/7	4 (0–13)	11.5 (8–17)	9 (8–12)
Sitoste-rol	0.269 (0.225–0.336)	0.286 (0.258–0.306)	0.257 (0.212–0.309	13.5 (12–30)	14.5 (11–32)	26.5 (11–32)	0/10	4/10*	9/10	0* (0)	0* (0–15)	9.5 (0–17)
Stigma-sterol	0.268 (0.206–0.312)	0.238 (0.169–0.272)	0.232 (0.164–0.286)	26 (11–31)	15* (8–31)	20 (11–28)	4/10	3/10*	2/9*	0 (0–9)	0* (0–8)	0* (0–10)
Cholestan-3-one	0.269 (0.185–0.337)	0.281 (0.214–0.331)	0.294 (0.255–0.342)	20 12–31)	11.5* (8–30)	26 (13–30)	2/10	1/10*	8/10	0 (0–8)	0* (0–3)	8 (0–15)
Cholest-4-en-3-one	0.271 (0.222–0.313)	0.239 (0.173–0.269)	0.245 (0.193–0.293)	17 (11–32)	11* (8–28)	12.5* (10–28)	2/10	2/9*	1/10*	0 (0–7)	0* (0–8)	0* (0–6)
?^2^ value	0.53	**14.86**	13.76	3.86	**17.84**	**17.14**	10.48	**20.23**	**22.53**	11.10	**28.42**	**29.07**

Kruskal-Wallis results are reported for each column, with critical probability after Bonferroni correction for three tests (lifespan, relative growth rate) = 0.016, and for six tests (reproductive indices) = 0.008, with statistically significant values indicated in bold; *indicates significant reduction compared to cholesterol (p<0.05).

**Figure 2:**
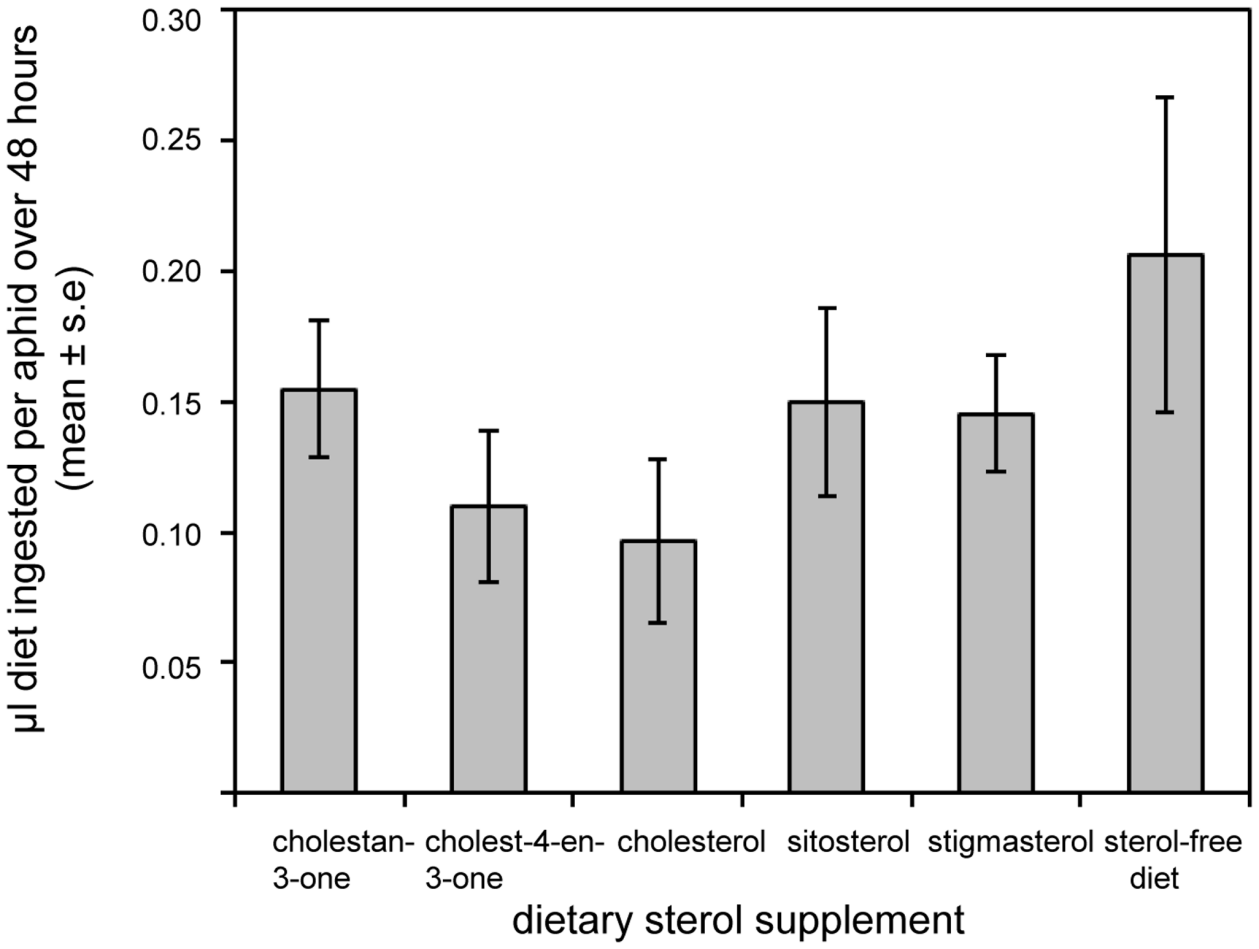
Volume of diet ingested by *Myzus persicae* on sterol-supplemented diets. The experiments quantified radioactivity in the honeydew of aphids fed on diet supplemented with ^14^C-inulin, which does not traverse the gut wall of these aphids.

To examine if lifespan and reproduction differences between the different sterol/steroid treatments might be attributed to differences in consumption, we examined food intake from diets containing 10 µg ml^−1^ of each test sterol/steroid. Individual aphids consumed 0.1–0.15 µl diet over a period of 48 hours (Figure 2), with no significant variation with diet composition (F_5,47_ = 1.150, *P* = 0.348).

### Interaction between Cholesterol and Cholest-4-en-3-one

This experiment investigated whether cholest-4-en-3-one, a steroid that did not promote aphid reproduction, influenced the effect of dietary cholesterol on aphid performance (Table 2). *M. persicae* was reared on diets containing different concentrations of cholesterol (0, 0.1 and 10 µg ml^−1^), with or without cholest-4-en-3-one (10 µg ml^−1^). Significant differences in reproductive output were observed across the six diets (Table 2) so specific statistical comparisons were made. First, as observed previously (Table 1), reproductive output was significantly higher on the high cholesterol diet (10 µg ml^−1^) compared to diet with 10 µg cholest-4-en-3-one ml^−1^. Next we tested if dietary cholest-4-en-3-one interacts with cholesterol use. Aphid reproductive output did not differ significantly between diets containing 10 µg cholesterol ml^−1^ and 10 µg cholesterol plus 10 µg cholest-4-en-3-one ml^−1^. Furthermore, supplementing low cholesterol diets (0.1 µg ml^−1^) with 10 µg cholest-4-en-3-one ml^−1^ did not rescue reproduction relative to the high cholesterol diet. We concluded that cholest-4-en-3-one does not interact with the effect of cholesterol on aphid performance.

**Table 2:** Aphid reproduction on diets containing cholesterol, plus or minus cholest-4-en-3-one.

Diet	Number of reproducingaphids/total	Number of offspring aphid^−1^Median (range)
Sterol-free	11/20*	1* (0–12)
Cholesterol (0.1 µg ml^−1^)	10/20*	0.5* (0–13)
Cholesterol (10 µg ml^−1^)	16/18	9 (0–17)
Cholest-4-en-3-one (10 µg ml^−1^)	4/19*	0* (0–11)
Cholesterol (0.1 µg ml^−1^) +cholest-4-en-3-one (10 µg ml^−1^)	7/18*	0.5* (0–15)
Cholesterol (10 µg ml^−1^)+cholest-4-en-3-one (10 µg ml^−1^)	14/20	7.5 (0–16)
χ^2^	**18.82**	**25.56**

Kruskal-Wallis results are reported for each column, with critical probability = 0.025 after Bonferroni correction for two tests. Statistically significant values of χ^2^ are shown in bold. * indicates a significant reduction compared to 10 µg cholesterol ml^−1^ (p<0.05).

### Aphid Performance on Sterol Mixes Reproducing Normal and Modified Tobacco

The final experiments tested the performance of *M. persicae* on chemically-defined diets containing a sterol mix representing the phloem sap of control and modified tobacco plants [10], at three total sterol/steroid concentrations. Aphid lifespan and reproductive output did not vary significantly between the two diets at each of the three total sterol/steroid concentrations tested but, after combining the datasets for the three concentrations, the reproductive indices were significantly reduced in aphids reared on the diet mimicking the modified plants relative to the control diet (Table 3).

**Table 3:** Aphid performance on diets containing sterol profiles that mimic the phloem sap of control tobacco, and modified tobacco.

Dietary sterol (µg ml^−1^)	Lifespan (days) Median (range)			Number of reproducing aphids			Number of offspring aphid^−1^		
			?^2^	number/total		?^2^	Median (range)		?^2^
	Control diet^1^	Modified diet^2^		Control diet^1^	Modified diet^2^		Control diet^1^	Modified diet^2^	
1	25 (10–38)	23.5 (10–30)	1.57	17/19 (89%)	14/20 (70%)	1.56	8 (0–17)	3 (0–12	3.33
5	26 (17–38)	24 (13–35)	0.82	14/15 (93%)	13/20 (65%)	2.08	9 (0–15)	5.5 (0–14)	3.25
10	26.5 (6–33)	23.5 (12–32)	3.04	17/18 (94%)	13/18 (72%)	1.91	9 (0–13)	6.5 (0–13)	1.54
Pooled concen-trations	26 (6–38)	24 (10–35)	4.65	48/52 (92%)	40/58 (69%)	3.88	9 (0–17)	5.5 (0–14)	**8.13**

One-tailed Kruskal-Wallis tests are applied because aphid performance is predicted to be higher on the control diet than modified diet: critical probability for lifespan = 0.013 after Bonferroni correction for 4 tests; critical probability for reproductive indices = 0.006 after Bonferroni correction for 8 tests. Statistically significant values of χ^2^ are indicated in bold.

1 Composition of control tobacco phloem sap: 99% cholesterol, 1% stigmasterol [9].

2 Composition of modified tobacco phloem sap: 85% cholest-4-en-3-one, 14% cholesterol, 1.1% cholestan-3-one [9].

## Discussion

The genetic basis of plant function is complex, such that single genes may have multiple pleiotropic consequences. For this reason, transgenes can affect a range of plant traits, including some that are not necessarily easy to predict from their specific function. The *choM* transgene in the modified tobacco plants used in this study has been demonstrated to mediate increased levels of ketosteroids in both leaf tissue and phloem sap, without any detectable impact on plant growth or seed production [6]–[10]. We cannot exclude the possibility, however, that the oxidase function of *choM* may have other effects on the physiology of the plant, either a consequence of the altered steroid profile or possibly oxidative side-reactions of the enzyme. For these reasons, it is vital to test whether the poor insect performance on plants that bear the *choM* transgene can be attributed directly to the ketosteroids in the phloem sap.

This study focused on aphids reared for a single generation on chemically-defined diets with different steroid supplements; the second generation aphids died as larvae on all diets, indicative of a dietary deficiency unrelated to sterol nutrition. Our data revealed significant variation in the capacity of both phytosterols and ketosteroids to support the reproductive output of *M. persicae*. Between the two phytosterols, sitosterol supported superior aphid performance to stigmasterol, and between the two ketosteroids, cholest-3-one was superior to cholest-4-en-3-one (Table 1). The biochemical basis for this variation in aphid response to different steroids is not understood, but may include differences in susceptibility of sitosterol and stigmasterol to aphid-mediated dealkylation, and insect capacity to modify the ketone at C3 to a hydroxyl group in different ketosteroids [1], [11].

The specific purpose of this study was to compare the performance of *M. persicae* reared on diets containing sterol and ketosteroids to their performance on the modified tobacco plants expressing similar phloem-mobile sterol/ketosteroids [10]. The aphid cultures on modified plants suffered high mortality, and all individuals born on the plant died as larvae [10]. This effect is indicative of strong toxic or antifeedant effects because sterol reserves within the insect body buffer aphids against dietary insufficiency of utilizable sterol over this period of time [12]. Individual ketosteroids had no antifeedant effect when supplied via chemically-defined diet (Table 2), and most individuals born on the single sterol/ketosteroid diets survived to adulthood with median aphid lifespan >7–10 days (with some individuals surviving to at least 28 days). Although aphids on diets with cholest-4-en-3-one as the sole steroid had depressed reproductive output relative to aphids on diets containing cholesterol, cholest-4-en-3-one did not depress aphid performance on diets that also contained at least 13% cholesterol (Table 2 and Table 3). This suggests that the impact of cholest-4-en-3-one can be modified by the availability of dietary cholesterol. The comparison of aphid performance on diets with sterol profiles matching the composition of plant phloem sap indicated that the steroid composition of the modified plant sap did not significantly affect lifespan and had small negative effects on reproductive output (Table 3). The discrepancies between the results obtained for diet- and plant-reared aphids cannot readily be attributed to concentration differences between phloem sap and the diets. Although their absolute concentration in the tobacco phloem sap remains to be determined, the phloem-mobile sterols of other plants attain 0.3–3 µg ml^−1^ [13], [14], which lies within the range (0.1–10 µg ml^−1^) adopted for our dietary analysis.

The most parsimonious interpretation of these results is that the ketosteroids are poorly utilized by the aphids, but not acutely toxic. This is especially true when ketosteroids are paired with a minimal amount of cholesterol. Consequently, the exceptionally poor performance of the aphids on modified tobacco plants [9] cannot be attributed exclusively to the ketosteroids. This result contrasts with the evidence that dietary cholestan-3-one causes a substantial reduction in developmental rate of the lepidopteran *Heliothis zea*, which feeds on bulk plant tissue, not plant sap [9]. It would, however, be premature to conclude that the different insects vary in their susceptibility to ketosteroids because the concentration of the dietary steroids used in the study of *H. zea* [9] was 170 µg ml^−1^, an order of magnitude greater than used in our study on *M. persicae*. These data suggest that the plant resistance mechanism in the modified plants containing the *choM* transgene may comprise a synergistic interaction between the ketosteroids and other plant constitutents that are absent from the diet. Further progress in elucidating the underlying mechanisms will depend on a greater understanding of the fate of ketosteroids ingested by the aphids, including the extent to which these compounds are assimilated across the gut wall and their subsequent accumulation or metabolic transformations in the insect tissues.

## Materials and Methods

### Experimental Sterols and Diets

The sterols/steroids were purchased from Sigma Chemical (St. Louis, MO, USA) or Steraloids Inc. (Newport, RI, USA); the other diet constituents were purchased from Sigma Chemical (St. Louis, MO, USA). The purchased sterols were tested for purity by HPLC against standards: cholesterol, stigmasterol, cholestan-3-one, and cholest-4-en-3-one were >99% pure, and sitosterol (from Sigma Chemical), which was 60% pure, and was brought to >99% purity by HPLC. Chemically-defined diets were prepared as described previously [15]. Each diet contained 0.15 M amino acids and 0.5 M sucrose, and sterols/steroids were added following published methods [11]. Briefly, they were dissolved in chloroform (1 mg ml^−1^), and added to diets at concentrations between 0.1 and 10 µg sterol ml^−1^.

### Experimental Aphids

The green peach aphid *Myzus persicae* Sulzer clone SB10/1 was derived from a single parthenogenetic female from a long-term laboratory colony maintained and cultured on preflowering tobacco (*Nicotiana tabacum*) cv Samsun. Routine cultures and all experiments were conducted at 20°C with 18L: 6D light regime at 100 µmol m^−2^ sec^−1 ^PAR. The experimental insects were larvae deposited onto sterol-free diet over 24 h by adult apterous aphids collected from routine culture on plants. When 2-days-old, the larvae were transferred individually to their test diet in 2.5 cm diam. cages, maintained at 75% relative humidity; all diets were changed every third day. For each treatment, 10 replicate larvae were reared singly. The weight of each larval aphid at day 2 and day 7 was determined to an accuracy of 1 µg,; for these two time periods, and the relative growth rate (RGR) for each insect was calculated as: RGR = [log_e_ (final weight/initial weight)]/number of days. The insects were monitored daily until death, and for each aphid lifespan and reproductive output was recorded.

### Feeding Rates

Food uptake by aphids was quantified by a published radioisotope technique, in which the non-permeant polysaccharide inulin, labeled with ^14^C, is included in the diet at a known concentration, and volume ingested is calculated from the amount of inulin recovered from the honeydew, as determined by ^14^C liquid scintillation counting [16], [17]. Preliminary experiments (data not shown) confirmed that <10% of ingested ^14^C was recovered from the body, indicating that, as in previous research, the aphid gut is impermeant to inulin. Twenty-five 7-day-old aphid larvae, reared from birth on a sterol-free diet, were transferred individually to a Perspex ring (3.5 cm diam., 0.5 cm height) with diet containing 16 µCi [^14^C] inulin ml^−1^ (Sigma), either supplemented with 10 µg non-radioactive sterol (cholesterol, sitosterol or stigmasterol), or no sterol. Honeydew produced by the aphid was deposited onto a 3.5 cm circle of absorbent paper (Nuc-wipes, National Diagnostic) placed under each ring. After 48 h, the paper circle was transferred to 5 ml Ecoscint (National Diagnostic) and counted in a scintillation counter (Beckman LS6500), with a preset ^14^C window and quench curve. The mean of three replicate aphids and paper circles on non-radioactive diets of the same formulation was subtracted from the experimental values. Control experiments confirmed that the radioactivity in the aphid carcass was consistently <10% of the radioactivity recovered from the honeydew, confirming that the inulin is not assimilated.

### Statistical Analysis

All data sets were checked for normal distributions by the Anderson Darling test, and homogeneity of variances by the Levine and Bartlett tests. All the aphid performance data (lifespan, reproduction, RGR) followed non-normal distributions, and were analyzed using nonparametric tests. Kruskal-Wallis tests were used for analysis of lifespan, number of offspring/aphid, and RGR; where significant differences were detected, and when there were more than 2 treatments, post-hoc comparisons were performed with a specified control treatment, using the Dunn Method for Joint Ranking [18]. The number of aphids reproducing was analyzed using Proportion tests, and Tukey-type multiple comparisons were performed to identify which treatments differed from one another [19]. Food uptake followed a normal distribution and was analyzed with ANOVA.
